# The representation of scientific research in the national curriculum and secondary school pupils’ perceptions of research, its function, usefulness and value to their lives

**DOI:** 10.12688/f1000research.7449.2

**Published:** 2016-02-12

**Authors:** Kay Yeoman, Laura Bowater, Elena Nardi

**Affiliations:** 1 School of Biological Sciences, University of East Anglia, Norwich, UK; 2 Norwich Medical School, University of East Anglia, Norwich, UK; 3 School of Education and Lifelong Learning, University of East Anglia, Norwich, UK

**Keywords:** Research Perception, Secondary School, Research-led Teaching, National Curriculum, National Curriculum for Science, Value, Career, Science Education, STEM

## Abstract

Young people’s views on what research is, how it is conducted and whether it is important, influences the decisions they make about their further studies and career choices. In this paper we report the analysis of questionnaire data with a particular focus on pupil perceptions of research in the sciences and of the scientific method. The questionnaire was a 25-item Likert Scale (1-5) distributed to seven collaborating schools. We received 2634 returns from pupils across key stages 3, 4 and 5. We also asked teachers to complete the questionnaire in order to explore how they thought their pupils would respond. We received 54 teacher responses. Statistically significant differences in the responses were identified through a chi-square test on SPSS. As what is being taught influences secondary pupil views on research we also consider how the term ‘research’ appears in the national curriculum for England and Wales and the three main English exam boards. The main theoretical construct that informs our analysis of the questionnaire data and the national curriculum is Angela Brew’s 4-tier descriptor of perceptions of research (domino, trading, layer, journey). We use this framework in order to map what, when and how research is presented to school pupils in England and Wales. We also use this framework in order to highlight and discuss certain pupil views that emerged from the questionnaire data and which indicate areas where curriculum and pedagogy intervention may be necessary: pupils seem less confident in their understanding of research as involving the identification of a research question; and, they often see research as a means to confirm one’s own opinion. They do however understand research as involving the generation of new knowledge and the collection of new data, such as interviews and questionnaires as well as laboratory work, field trips and library searches and they appear relatively confident in their statements about their ability to do research, their school experiences of research and the importance of research in their future career choice.

## Introduction

Research is a process that occurs in all disciplines and a society’s knowledge economy is reliant on it. The United Kingdom (UK) is very successful in the quantity and quality of science it produces – it is ranked first in field-weighted citation impact. Despite having only 4.1% of the world’s researchers, it accounts for 15.9% of the world’s most highly cited papers ( International Comparative Performance of the UK Research Base, 2013). A good example of research benefiting economy is highlighted in a report by the Institute of Food Research, which is funded by the Biological and Biotechnological Science Research Council (BBSRC). The report demonstrates that for every £1 invested in research, £8 is returned to the UK economy ( Brookdale Consulting, 2013). To ensure that the UK maintains economic prosperity in the future, future generations need to engage with science, technology, engineering and mathematics (STEM) subjects. Considerable effort is being made to raise the profile of these subjects in secondary schools in order to encourage pupils to take up these subjects at A level and beyond. It has been noted that, as societies become more reliant on science and technology, fewer school-aged children are choosing science and technology as a career path ( Donghong & Shunke, 2010). Clearly, this is a concern: as research by the UK science council suggests in 2017 over 58% of jobs will require skills in STEM subjects ( http://www.score-education.org/media/3668/report.pdf).

Research can be defined in many different ways. For example, the Oxford English Dictionary (OED) defines research as “ *Systematic investigation or inquiry aimed at contributing to knowledge of a theory, topic, etc., by careful consideration, observation, or study of a subject*.” (OED Online http://www.oed.com/view/Entry/163432?rskey=RKm0Mc&result=1#eid). Redman & Mory (1923) defined research as “ *systematised effort to gain new knowledge*”. The four UK research councils as part of the Research Excellence Framework (REF) define research as “ *a process of investigation leading to new insights, effectively shared*” ( http://www.ref.ac.uk/pubs/2011-02/).

The concept of a ‘process of investigation’ is embedded within the philosophy of the scientific method. The concept first emerged from Francis Bacon’s ideas of inductive reasoning and was adopted by the Royal Society in the 1660s as a method to promote systematic investigation ( Purver, 2013). One definition of the scientific method as a series of discrete steps which could be used for teaching the scientific method in secondary schools originated with Keeslar in 1945 ( Keeslar, 1945). Keeslar designed a questionnaire which listed statements to do with elements of the scientific method. This questionnaire was sent to 22 scientists at the University of Michigan, who then ranked/agreed/disagreed with the statements. The responses were analysed by assigning a relative numerical value to each statement on a 200 point scale, by using a formula designed by Keeslar; a series of 12 steps emerged which formed the basis of the schematics that are taught in schools worldwide ( McComas, 1998).

**Figure 1.**
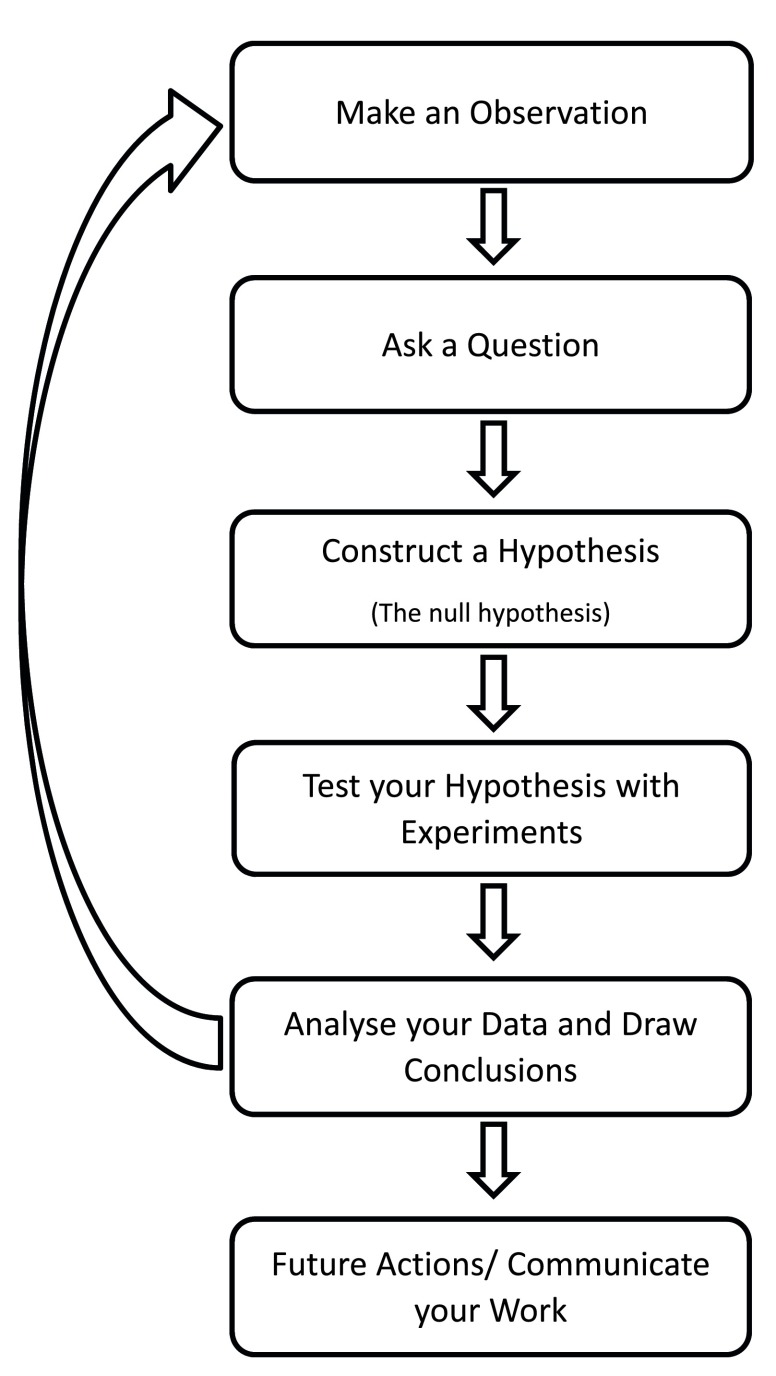
A schematic of the scientific method.

However, within the scientific research community, the scientific method is applied in different ways and not always in accordance with the rigid, linear investigation schematic that is often portrayed in text books ( Figure 1). New researchers learn how to conduct research through participation in scientific studies under the guidance of experienced researchers ( McComas, 1998; Sarma, 2014).

In 2001 Angela Brew conducted a phenomenographic study into how research was experienced by established researchers. Her investigation uncovered four different ways in which research is perceived:

1. **Domino variation**-where research is viewed as comprising tasks, events, things, activities, problems, techniques, experiments, issues, ideas or questions.
2. **Trading variation**-where research is seen as product and social phenomenon, e.g. in terms of publications, grants and social networks.
3. **Layer variation**-where research brings to light ideas, explanations and truths.
4. **Journey variation**-where the activities in which the researcher engages enables them to grow or transform.

We note that there are very few studies that have looked at the perceptions of research by secondary school pupils and the value they place on research in relation to their future careers. Studies that have been conducted in this research area have focused on postgraduate students in higher education ( Meyer *et al.*, 2005) or experienced researchers ( Brew, 2001). We see our project as a potential contributor to this under-researched area by exploring how pupils currently conceive research and science. We ask the following questions:

- How is the term research used in the national curriculum, the national curriculum for science in England and in examination board specifications?
- Do pupils consider research to be a process or an output?
- Do pupils consider research to be challenging?
- Do pupils consider research to be valuable to them for their future?
- Do pupils consider that they do research within the school environment?
- How do teachers think pupils perceive research?

## Materials and methods

A questionnaire was designed in a series of research team meetings in the early months of the study. Starting from one of the widely-used and reliability-tested Fennema-Sherman Mathematics Attitudes Scales ( Fennema & Sherman, 1976; Wikoff & Buchalter, 1986), 25 items were constructed around the four themes *who does research*, the *value of research*, the *process of research,* and *myself and research* (6, 4, 9 and 6 items respectively). Attention was given to the inclusion of both positive and negative statements. Seven schools located in East Anglia participated ( Table 1). The questionnaire was piloted to about 600 pupils in School C and refined further prior to its use, with randomised item order, with the large cohort of about 6,000 pupils. For the questionnaire see the supplementary information. The questionnaire was distributed to and collected from school pupils across all year groups by school teachers. Pupils completed the questionnaire during their morning registration period. We received 2634 responses. The responses from key stage 5 pupils were from a broad spectrum of pupils studying a wide variety of subjects. The questionnaires were scanned by the data collection company Kendata ( http://www.kendata.com/), and an Excel database of responses was compiled and then imported into SPSS version 22. For statistical analysis the year groups were collated according to key stage ( Table 2) and the Likert scale was coded in SPSS as strongly agree/agree (1); neither agree not disagree or unsure (2) and disagree/strongly disagree (3). The data was analysed using Pearson’s chi-square test. It was recognised that large data sets can yield small p values; so to increase the robustness of the analysis the probability was set at <0.001 in order to be deemed significant.

**Table 1.** School type and Ofsted rating of schools taking part in the study. ^1^Rating is as determined by the Office for Standards in Education, Children’s Services and Skills (Ofsted).

School	Type	Description	Key Stages Taught	Current Ofsted rating ^1^
A	State	Small, mixed rural location	KS3 and 4	Good
B	State	Large, mixed, town location	KS3, 4 and 5	Requires Improvement
C	State (Academy status)	Large, mixed, city location	KS3, 4 and 5	Requires Improvement
D	State	Large, mixed, coast location	KS5	Good
E	Independent	Small, mixed, city location	KS3, 4 and 5	Outstanding
F	State (Academy status)	Large mixed, rural location	KS3, 4 and 5	Special Measures
G	State (Academy status)	Large, mixed town location	KS3, 4 and 5	Good

**Table 2.** Number of pupil responses in terms of gender and key stage.

	Gender		Key Stage			School Type	
	Male	Female	3 (aged 11–14) Years 7, 8 and 9	4 (aged 14–16) Years 10 and 11	5 (aged 16–18) Years 12 and 13	State	Independent
Sample (n)	1134	1259	928	845	861	2200	434

The questionnaire was converted into an online form, and teachers were asked to fill it in according to how they thought their pupils would respond. The teacher sample size was 54, with 49 from state schools and 5 from an independent school. The teachers who responded were from different disciplines across the sciences and humanities. In order to compare the pupil and teacher data a randomised stratified sample (n=54) of the complete pupil data set (n=2634) was compared against the data from the teachers. The data was analysed using Pearson’s chi-square test. Due to the smaller sample size [n=108] compared to the total pupil questionnaire data [n=2634] the probability was set at <0.01 in order to be deemed significant.

The questionnaire on research perception was distributed to the seven schools, and was provided to pupils by form tutors during the morning registration period. There were a total number of 2634 returns, with the sample split in terms of gender and key stage as shown in Table 2.

### Ethics statement

All phases of the research have been approved by the School of Education’s Research Ethics Committee (EDU-REC). Consent for participation in the project was secured through the distribution of information sheets and collection of signed consent forms from teachers, parents and pupils over the age of 16. As a complement to parental consent pupils under the age of 16 signed assent forms. Participation in the study took place during school time (either during lessons or outside lessons) and as part of the students’ learning experience about research. The teachers encountered no problems as their schools are official partners of the project and participation implied minimal interference with one normal school day and was carried out with adequate notice. Across all phases of the study, including the analysis of the data and the dissemination of the findings, confidentiality, anonymity and right of withdrawal rules have applied throughout. We note that EDU-REC complies with the British Educational Research Association’s Revised Ethical Guidelines for Educational Research. The research team carried out the research in awareness of the relevant sections of the Data Protection Act (1998): http://www.hmso.gov.uk/acts/acts1998/19980029.htm and Freedom of Information Act (2005).

The purpose and procedures of the research, and the potential benefits and costs of participating (e.g. the amount of their time involved) were fully explained to teachers, parents and pupils at the outset. The full identity of all members of the research team was revealed to potential participants. No pressure was placed on any individual or institution to participate in the study and the treatment of no individual was in any way prejudiced if they chose not to participate in the project. Schools, teacher and parents were provided with the UEA contact details of team members ( *not* personal contact details) in order that they could make contact in relation to any aspect of the research, should they wish to do so. We notified participants that complaints could be made to the EDU Head of School. Participants were made aware that they may freely withdraw from the project at any time without risk or prejudice. Research activities were carried out with regard for mutually convenient times and negotiated in a way that seeks to minimise disruption to schedules and burdens on teachers, pupils and their parents. The views of all participants in the research were respected. The team was alert and sensitive to any prejudice that may emerge from differences relating to age, culture, disability, race, sex, religion and sexual orientation, when planning, conducting and reporting the research. The original hard copies of the questionnaires are kept in a safe and secure location and are being used purely for the purposes of the research project (including dissemination of findings). No-one other than research colleagues have access to any identifiable raw data collected. Participants have been informed that they have the right of access to any data pertaining to them. All necessary steps have been taken to protect the privacy and ensure the anonymity and non-traceability of participants – e.g. by the use of pseudonyms, for both individual and institutional participants, in any written reports of the research and other forms of dissemination.)

## Results

Complete pupil data set The complete data set of all the pupil returns from seven different participating schools. The data is anonymised. The question number can be related to the actual question by referring to the questionnaire in the supplementary information. The Likert scale is as follows; 1:strongly disagree; 2:disagree; 3:neither agree nor disagree or unsure; 4:agree; 5:strongly agree. Click here for additional data file. Copyright: © 2016 Yeoman K et al. 2016 Data associated with the article are available under the terms of the Creative Commons Zero "No rights reserved" data waiver (CC0 1.0 Public domain dedication).

Compiled teacher:pupil data set The complete data set of the teacher responses with a random stratified sample of the pupil responses. The data is anonymised. The question number can be related to the actual question by referring to the questionnaire in the supplementary information. The Likert scale is as follows; 1:strongly disagree; 2:disagree; 3:neither agree nor disagree or unsure; 4:agree; 5:strongly agree. Click here for additional data file. Copyright: © 2016 Yeoman K et al. 2016 Data associated with the article are available under the terms of the Creative Commons Zero "No rights reserved" data waiver (CC0 1.0 Public domain dedication).

## The representation of research in the national curriculum for science in England and the examination board specifications

Two researchers (the first two authors) undertook the mapping of the national curriculum for Science in England (NCSE) for key stage 3 ( https://www.gov.uk/government/uploads/system/uploads/attachment_data/file/335174/SECONDARY_national_curriculum_-_Science_220714.pdf), key stage 4 ( https://www.gov.uk/government/uploads/system/uploads/attachment_data/file/381380/Science_KS4_PoS_7_November_2014.pdf) and key stage 5 ( https://www.gov.uk/government/uploads/system/uploads/attachment_data/file/446829/A_level_science_subject_content.pdf) to Brew’s framework independently. We used the documents pertaining to teaching in the 2013–14 academic year, as this was when the data was collected. Initially, a discussion was held to ensure that both researchers held a shared understanding of each component of the framework. Each individual learning outcome of key stage 3, 4 and 5 was assigned as trading, journey, domino and variation. Assignment of learning outcomes by both researchers was compared and, where disagreement arose, discussion was held with a third researcher until a consensus was reached. The national curriculum (NC, across areas of study) was also scrutinised for mention of the word ‘research’, or any phraseology that could be identified as referring to research ( https://www.gov.uk/government/uploads/system/uploads/attachment_data/file/335116/Master_final_national_curriculum_220714.pdf).

## The national curriculum and the national curriculum for science in England

As the research in this paper was conducted in England, references to curriculum are restricted in this region. The NC provides all local authority-maintained schools in England “ *the programmes of study and attainment targets for all subjects, at all key stages*” (p.13) ( https://www.gov.uk/government/uploads/system/uploads/attachment_data/file/335116/Master_final_national_curriculum_220714.pdf). The key stages (KS) are described in Table 3 and contain key learning milestones that should be delivered to pupils across the breadth of taught subjects and disciplines. It is acknowledged that independent schools, free schools and academies do not need to follow the NC. The national curriculum for science in England (NCSE) applies to Biology, Chemistry and Physics and at key stage 5 (KS5) it also includes psychology.

**Table 3.** The school structure in England with associated qualifications.

Key Stage	Ages	School Years	Qualification
**1**	5–7	1 and 2	-
**2**	7–11	3, 4, 5 & 6	-
**3**	11–14	7,8 & 9	-
**4**	14–16	10 & 11	GCSE
**5**	17–19	12 & 13	A level

An initial analysis of the NC demonstrates that science is placed in high regard and it is felt to be of importance to society. There is also a desire that pupils appreciate this importance: “ *Science (..) is vital to the world’s future prosperity and all pupils should be taught essential aspects of the knowledge, methods, processes and uses of science*” (p.168). A key aim is pupils should “ *develop understanding of the nature, processes and methods of science through different types of science enquiries that help them to answer scientific questions about the world around them*” (p.168) ( https://www.gov.uk/government/uploads/system/uploads/attachment_data/file/335116/Master_final_national_curriculum_220714.pdf). The NCSE has also established a key aim for pupil attainment with a main outcome of the curriculum in key stages 4 and 5 that pupils should appreciate and establish an optimistic and positive view of the role and impact of science in providing solutions to societal problems “ *pupils should appreciate the achievements of science*” (p.3); ‘ *the role of science in understanding the causes of and solutions to some of the challenges facing society*” (p.4) ( https://www.gov.uk/government/uploads/system/uploads/attachment_data/file/381380/Science_KS4_PoS_7_November_2014.pdf). The NCSE at key stage 5 provides a strong, positive vision that pupils should acknowledge science as a solution provider on behalf of society. They are required to understand ‘ *how society makes decisions about scientific issues* and ‘ *how sciences contribute to the success of the economy*’ (p.3) ( https://www.gov.uk/government/uploads/system/uploads/attachment_data/file/446829/A_level_science_subject_content.pdf).

The requirements of a future society with a workforce with skills in STEM is stressed in the curriculum. In key stage 4 (KS4) the NCSE states that teaching should “ *establish the basis for a wide range of careers*”(p.3) ( https://www.gov.uk/government/uploads/system/uploads/attachment_data/file/381380/Science_KS4_PoS_7_November_2014.pdf). The KS5 documentation lists a key aim to “ *develop (their) interest in and enthusiasm for the subject, including developing an interest in further study and careers associated with the subject*” (p.3) ( https://www.gov.uk/government/uploads/system/uploads/attachment_data/file/446829/A_level_science_subject_content.pdf).

The NCSE was mapped against the Brew (2001) framework and this is shown in Table 4.

**Table 4.** The NCSE learning outcomes at different key stages mapped against the Brew (2001) framework.

Learning outcomes	Key Stage 3	Key Stage 4	Key Stage 5	Mapping to the Brew Framework
The development of scientific thinking				
Understand that scientific methods and theories develop over time	✓	✓	✓	Layer Variation
Understand that science progresses through a cycle of hypothesis, practical experiments, observation, theory development and review	✕	✓	✓	Domino Variation
Use a variety of concepts and models to develop scientific explanations and understanding	✕	✓	✓	Domino Variation and Layer Variation
Understand that change is driven by interactions between different objects and systems over distance and time	✕	✓	✓	Layer Variation
Understand the assumption that every effect has one or more causes	✕	✓	✓	Layer Variation
Appreciate the power and limitations of science and consider ethical issues	✕	✓	✓	Layer and Journey Variation
Explain everyday and technological applications of science; evaluate personal social, economic and environmental implications	✕	✓	✓	Layer and Journey Variation
Evaluate risks in practical sciences	✓	✓	✓	Domino variation
Evaluate risks of science in the wider societal context	✕	✓	✓	Domino variation
Understand the importance of publishing results and peer review	✕	✓	✓	Trading and Journey Variation
Recognise the importance of communicating results to a range of audiences	✕	✓	✓	Trading and Journey Variation
Develop the use of vocabulary and language	✓	✓	✓	Domino variation
Understand that quantitative analysis is a central element of theories and scientific methods of inquiry	✕	✓	✓	Domino Variation
Communicate the scientific rationale for investigations, including methods used, findings & conclusions	✕	✓	✓	Trading variation
Communicate research using paper based and electronic reports and presentations	✕	✓	✓	Trading and Journey Variation
Develop interest and enthusiasm, including interest in further study and careers associated with the subject	✕	✕	✓	Journey Variation
Develop essential knowledge and understanding of different areas of the subject and how they relate to each other	✕	✕	✓	Journey Variation
Experimental skills and strategies				
Use science to help develop hypothesis	✕	✓	✓	Domino variation
Make predictions using scientific knowledge and understanding	✓	✓	✓	Domino variation
Ask questions and develop a line of enquiry based on observation and prior knowledge and experience	✓	✓	✓	Domino variation
Select, plan and undertake appropriate types of scientific enquiry to test predictions including the use of variables	✓	✓	✓	Domino variation
Apply knowledge of a range of techniques, apparatus and materials to select those appropriate for fieldwork and experiments	✕	✓	✓	Domino variation
Use appropriate techniques, apparatus and materials in field and laboratory work including issues of health and safety	✓	✓	✓	Domino variation
Make and record observations and measurements using a range of methods	✓	✓	✓	Domino variation
Evaluate the reliability of methods and suggest improvements	✓	✓	✓	Domino variation
Apply suitable sampling techniques	✓	✓	✓	Domino variation
Analysis and Evaluation				
Present observations and data appropriately	✓	✓	✓	Domino variation
Translate data from one form to another	✕	✓	✓	Domino variation
Apply mathematical concepts and calculate results	✓	✓	✓	Domino variation
Carry out statistical analysis	✕	✓	✓	Domino variation
Represent distributions of results and make estimations of uncertainty	✕	✓	✓	Domino variation
Interpret observations and data to identify patterns and draw conclusions	✓	✓	✓	Domino variation
Present reasoned explanations in relation to predictions and hypothesis	✓	✓	✓	Layer Variation
Pay attention to objectivity and concern for accuracy, precision, repeatability and reproducibility	✓	✓	✓	Domino Variation
Evaluate data with respect to sources of error	✓	✓	✓	Domino variation
Resolve conflicting evidence	✕	✕	✓	Domino and Layer Variation
Identify further questions arising from results	✓	✓	✓	Domino variation

The analysis presented in Table 4 indicates that the NCSE maps to all aspects of the Brew (2001) framework. Thirty seven separate learning outcomes were identified across KS 3, 4 and 5; 68% of them map onto the domino variation; 22% map to layer and 19% to journey, and only 11% map to trading variation ( Table 4). The outcomes linked to ‘experimental skills and strategies’ and ‘analysis and evaluation’ are entirely dominated by the domino variation. This is not surprising as this variation describes research as activity, event, problems, technique and experiment. Learning outcomes under the ‘development of scientific thinking’ are more complex, and have examples mapped to layer variation (bringing to light ideas, explanations and truths), trading variation (research as product and social phenomenon) and journey variation (growth and transformation).

There are some differences across the different key stages. Learning outcomes that map to journey variation are not apparent at KS3, but do appear at KS4 (5 outcomes) and KS5 (7 outcomes). There are two examples of learning outcomes linked to layer variation at KS3, but this increases through KS4 (7 outcomes) to KS5 (8 outcomes). There are no trading variation outcomes at KS3, but there are four outcomes linked to this variation at both KS4 and 5.

STEM disciplines require and depend upon research skills and the NCSE describes a series of key learning outcomes, which are clearly part of a process of investigation and map to domino variation:

1. Ask questions and make predictions using scientific knowledge.
2. Carry out appropriate scientific enquiries to test predications.
3. Record observations and measurements and apply sampling techniques.
4. Present and interpret observations and data.
5. Present explanations in relation to predictions and hypothesis.
6. Identify further questions arising from results.

While the steps of the ‘scientific method’ are referred to within the NC, the actual term ‘scientific method’ is not present. Instead the phrase “ *working scientifically*” is used to describe “ *the key features of science enquiry, so that pupils learn to answer relevant scientific questions*” (p.169) ( https://www.gov.uk/government/uploads/system/uploads/attachment_data/file/335116/Master_final_national_curriculum_220714.pdf).

The word ‘research’ is not used at all in the two documents for KS 3 or 4. The concept promoted by Redman & Mory (1923) where research is defined as the ‘ *systematised effort to gain new knowledge*’ and the definition from the REF with research as “ *a process of investigation leading to new insights, effectively shared*” is not explicitly stated within the NCSE although it is suggested that pupils should “ *use scientific theories and explanations to develop hypotheses*” (p.5) and “ *interpret observations and other data including identifying patterns and trends, making inferences and drawing conclusions*” (p.6) ( https://www.gov.uk/government/uploads/system/uploads/attachment_data/file/381380/Science_KS4_PoS_7_November_2014.pdf). Within the KS5 document the word ‘research’ is specifically linked to psychology (rather than to biology, physics or chemistry) students must develop knowledge and “ *understanding of research in psychology*” (p.16) ( https://www.gov.uk/government/uploads/system/uploads/attachment_data/file/446829/A_level_science_subject_content.pdf). The word ‘research’ can also be found in Appendix 5 of the key stage 5 NCSE that states practical work undertaken by students throughout the A level syllabus should include ‘research and referencing’. This includes “ *the use of online and offline research skills including websites, textbooks and other printed scientific sources of information” and “correctly cite sources of information”* (p.20) ( https://www.gov.uk/government/uploads/system/uploads/attachment_data/file/446829/A_level_science_subject_content.pdf).

## The examination board specifications

Independent schools, free schools and academies do not need to follow the NCSE. However all schools in England offer qualifications through the three major exam boards in England: the Assessment and Qualifications Alliance (AQA), Edexcel (Pearson-London Examiners) and Oxford, Cambridge and Royal Society of Arts & Manufactories Examinations (OCR). These exam boards offer a range of qualifications, including the General Certificate of Secondary Education (GCSE), the Business and Technology Education Council (BTEC) and the General Certificate of Education (GCE). Thus we thought it important to look at the specification of qualifications from different exam boards to see how the term ‘research’ is used within this documentation. We focussed the investigation onto GCSE and GCE qualifications in biology as an example as the schools in this study all offer these courses.

At GCSE level the pupils are expected to consider evidence from different areas of scientific research, as shown by statements that include “ *explain how new evidence from DNA research and the emergence of resistant organisms supports Darwin’s theory*” (p.20) ( http://www1.edexcel.org.uk/science2011/GCSE_Biology.pdf) as well as to think about the “ *the social and ethical issues concerning the use of stem cells from embryos in medical research and treatments*” (p.39) ( http://filestore.aqa.org.uk/subjects/AQA-BIOL-W-SP-14.PDF). The OCR specification also clearly links the term ‘research’ to fact-finding, e.g. *“research diabetes and how it can be managed’ (p.24) and ‘research the work of John Ray and Carl Linnaeus in developing a modern classification system*” (p.30) ( http://www.ocr.org.uk/Images/82545-specification.pdf).

It transpires that in all the examination boards the controlled assessment requires the use of research, but the term is linked to secondary research, which can include extracts from books and websites. Students can carry out secondary research in a library or at home ( http://www1.edexcel.org.uk/science2011/GCSE_Biology.pdf). As part of the controlled assessment pupils “ *plan and carry out an investigation to collect primary data to test their hypothesis*” (p.116) ( http://www.ocr.org.uk/Images/82545-specification.pdf) but the term ‘research’ is not linked to this activity, but only to the former ‘fact finding’ part of the controlled assessment. Thus in these GCSE specifications investigation and research is split. The actual practical work is termed ‘investigation’, fact finding leading up to this is termed ‘research’.

At GCE level the term ‘research’ is used for evidence of practical work and as part of practical competency “ *uses appropriate software and/or tools to process data, carry out research and report findings*” (p.38) ( http://qualifications.pearson.com/content/dam/pdf/A Level/biology-b/2015/specification-and-sample-assessment-materials/9781446914533_GCE2015_A_BIOLOGYB for web.pdf). As with GCSE it is linked to fact finding “ *use online and offline research skills including websites, textbooks and other printed scientific sources of information*” (p.10) ( http://www.ocr.org.uk/Images/171736-specification-accredited-a-level-gce-biology-a-h420.pdf). The OCR specification now has a ‘research skills’ element to their practical portfolio which consists of the following:

- Apply investigative approaches
- Use online and offline research skills
- Correctly cite sources if information

Within the AQA GCE biology specification the term research is only mentioned under practical mastery, “ *carry out research and report findings*” (p.75) ( http://filestore.aqa.org.uk/subjects/specifications/alevel/AQA-2410-W-SP-14.PDF). The information presented here on the use of the term ‘research’ in qualification specification corresponds well to the use of ‘research’ in the NCSE.

The application of the term research in these different scenarios, on the one hand linking research to cutting edge scientific knowledge ‘embryonic stem cells’ but also linking it to basic ‘fact-finding’ at GCSE and GCE leads to a confusion over what research really is, which is evident in this paper.

We now present the questionnaire data on pupil perceptions of: what constitutes research; their experience and ability in research; and, their appreciation for research.

## The questionnaire data on pupil perceptions of research

**Figure 2.**
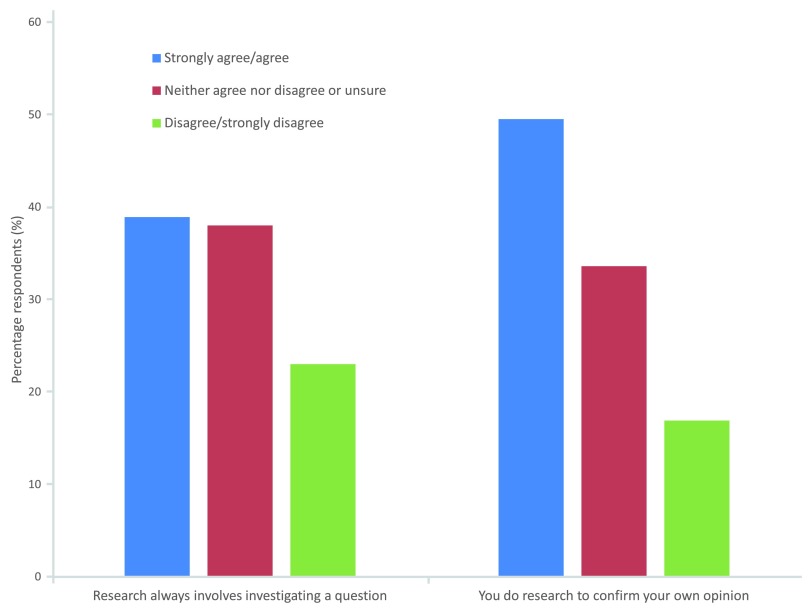
Percentage of pupil responses to the statements ‘research always involves investigating a question’ and ‘you do research to confirm your own opinion’. There was no significant difference with respect to gender (n=2362, *p*=0.002) or KS (n=2585, *p*=0.002) to the statement ‘research always involves investigating a question’. There was no significant difference with respect to gender (n=2355, *p*=0.3) or KS (n=2576, *p*=0.04) to the statement ‘you do research to confirm your own opinion’.

A fundamental part of the research process is the establishment of the research question. The NCSE at key stage 3 clearly indicates that students are expected to “ *ask questions and develop a line of enquiry based on observation and prior knowledge and experience*” (p.4) ( https://www.gov.uk/government/uploads/system/uploads/attachment_data/file/335174/SECONDARY_national_curriculum_-_Science_220714.pdf). To explore pupils’ perceptions of using questions within research and science investigation, Figure 2 shows the responses to the statement ‘research always involves investigating a question’. The response indicates that pupils were unclear that research should begin this way, only 38.8% strongly agreed or agreed with the statement. There was no significant difference in responses with regard to either gender or KS (χ ^2^[2, N=2362] 12.26, *p*=0.002) and (χ ^2^[4, N=2585] 16.80, *p*=0.002) respectively. This suggests that the perception of the importance of posing research questions did not increase as students gained more science investigation experience through their education. When teachers were asked how their pupils would respond to the statement there was no statistical difference in how the pupils responded and how the teachers thought they would respond, (χ ^2^[2, N=108] 4.54, *p*=0.1). The NCSE clearly indicates there is a requirement for pupils in secondary education to “ *ask questions*” in relation to scientific investigation (p.4) ( https://www.gov.uk/government/uploads/system/uploads/attachment_data/file/335174/SECONDARY_national_curriculum_-_Science_220714.pdf). The data presented here potentially indicates an issue with how this aspect of scientific inquiry and scientific process occurs in the school environment.

The scientific method requires the researcher to minimise bias. In addition the NCSE indicates that pupils should “ *pay attention to objectivity and concern for accuracy*” (p.4). Figure 2 shows that substantial number of pupils (50%) strongly agreed or agreed that you do research to confirm your own opinion. There was no significant difference in responses according to either gender or KS (χ ^2^[2, N=2355] 6.40, *p*=0.04) and (χ ^2^[4, N=2576] 4.78 *p*=0.3) respectively, indicating that this does not change with increasing research experience. There was also no statistical difference in how the pupils responded and how the teachers thought they would respond, (χ ^2^[2, N=108] 0.63, *p*=0.73).

Redman & Mory (1923) define research as ‘ *systematised effort to gain new knowledge*’. In addition the NCSE suggests that pupils should “ *use scientific theories and explanations to develop hypotheses*” (p.5) and “ *interpret observations and other data including identifying patterns and trends, making inferences and drawing conclusions*” (p.6) ( https://www.gov.uk/government/uploads/system/uploads/attachment_data/file/381380/Science_KS4_PoS_7_November_2014.pdf). However no clear learning outcome is provided that asks pupils to understand that scientific inquiry or research is a systemised effort to gain new knowledge. When we investigated pupils understanding of this with ‘the main purpose of research is to generate new knowledge’ more than 70% of pupils across all key stages strongly agreed/agreed with the statement. There was no significant difference is responses across KS (χ ^2^[4, N=2577] 4.18, *p*=0.43) or gender (χ ^2^[2, N=2356] 14.7, *p*=0.001). There was also no statistical difference in how the pupils responded and how the teachers thought they would respond, (χ ^2^[2, N=108] 2.26, *p*=0.32).

**Figure 3.**
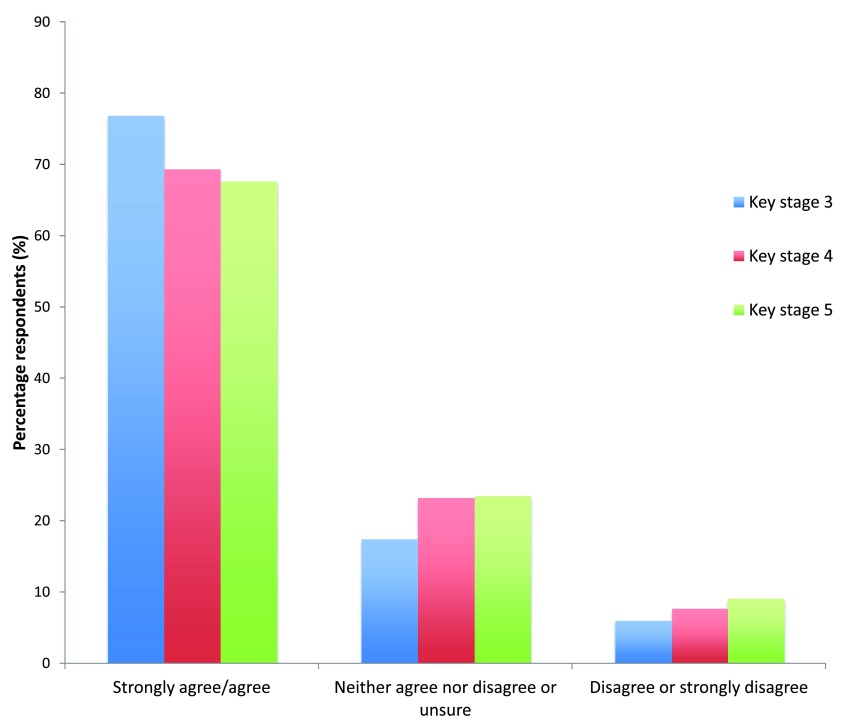
Percentage pupil responses across key stage to the statement ‘research involves collecting new data’. There was no significant difference in response with respect to gender (n=2356, *p*=0.001), but there was a significant difference across KS (n=2577, *p*<0.001).

Pupils were asked if research involves collecting new data ( Figure 3). There was no significant difference in the way in which males and females responded to the statement ‘research involves collecting new data’ (χ ^2^[2, N=2356] 14.1, *p*=0.001). However, there was a significant difference across KS (χ ^2^[4, N=2577] 22.16, *p*<0.001) with more pupils from KS3 (76.7%) strongly agreeing/agreeing with this statement than KS4 (69.2%) or 5 (67.5%). This could reflect a greater understanding at KS5 of how existing research data can be combined together and re-used in meta-analysis. This suggests a more sophisticated view of research which grows with experience. Many of the KS5 pupils in the schools who took part in this study have the opportunity to do an extended project qualification (EPQ) which would allow for this more nuanced understanding. These projects were discussed in structured interviews (data not presented as part of this paper). Overall though, pupils were more likely to strongly agree/agree with this statement (71.2%). There was no statistical difference in how the pupils responded and how the teachers though they would respond, (χ ^2^[2, N=108] 0.30, *p*=0.86).

**Figure 4.**
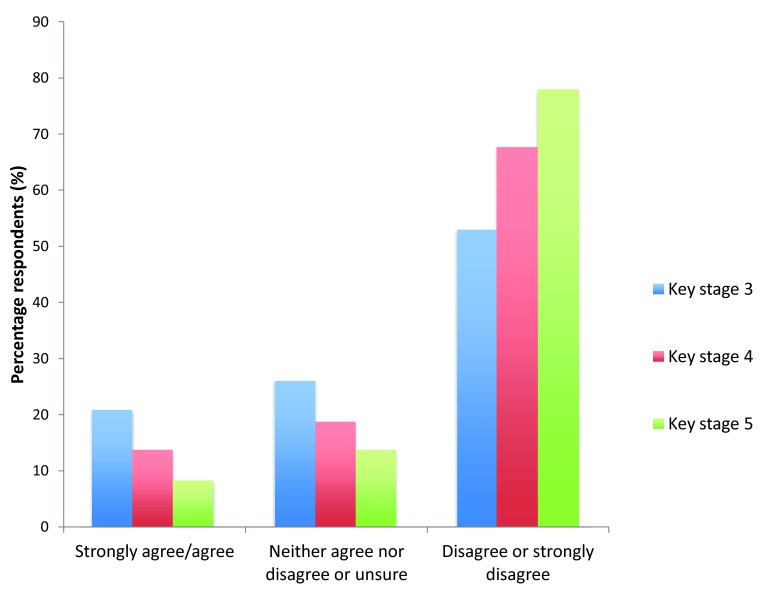
Percentage pupil responses across key stage to the statement ‘research is carried out solely through experiments in a laboratory’. There was no significant difference in response with respect to gender (n=2360, *p*=0.02), but there was a significant difference across KS (n=2577, *p*<0.001).

The NCSE states that school pupils should “ *select, plan and undertake appropriate types of scientific enquiry to test predictions including the use of variables and use appropriate techniques, apparatus and materials in field and lab work including issues of health and safety*” (p.4) as well as “ *make and record observations and measurements using a range of methods*” (p.4) ( https://www.gov.uk/government/uploads/system/uploads/attachment_data/file/335174/SECONDARY_national_curriculum_-_Science_220714.pdf). In Figure 4 pupils show an understanding that research could be conducted in areas other than a laboratory. There was no significant difference in the way in which males and females responded to the statement ‘research is carried out solely through experiments in a laboratory’ (χ ^2^[2, N=2360] 7.74, *p*=0.02). There was however a significant difference across KS (χ ^2^[4, N=2581] 124.97, *p*<0.001) with more pupils from KS5 (77.9%) disagreeing/strongly disagreeing with this statement than KS4 (67.5%) or KS3 (52.9%), again hinting at the greater experience of research methods and techniques as pupils move through the key stages. There was no statistical difference in how the pupils responded and how the teachers thought they would respond, (χ ^2^[2, N=108] 0.37, *p*=0.83).

**Figure 5.**
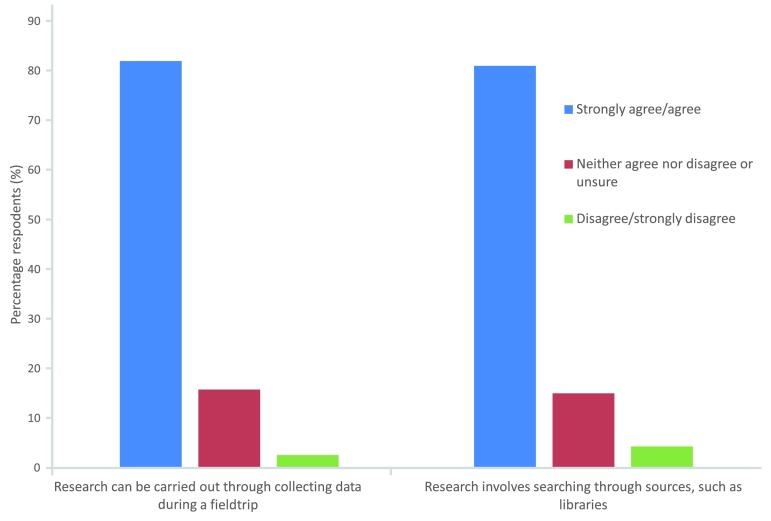
Percentage of pupil responses to the statements ‘research can be carried out through collecting data during a field trip’ and ‘research involves searching through sources such as libraries’. There was no significant difference with respect to gender (n=2368, *p*=0.01) or KS (n=2590, *p*=0.03) to the statement ‘research can be carried out through collecting data during a field trip’. There was no significant difference with respect to gender (n=2349, *p*=0.02) or KS (n=2538, *p*=0.69) to the statement ‘research involves searching through sources such as libraries’.

When asked to respond to the statement ‘research can be carried out through collecting data during a field trip’, there was no difference in response according to gender or across KS (χ ^2^[2, N=2368] 8.91, *p*=0.01) and (χ ^2^[4, N=2590] 10.85, *p*=0.03) respectively ( Figure 5). The majority of respondents strongly agree or agreed with this statement (82.1%). There was no statistical difference in how the pupils responded and how the teachers thought they would respond, (χ ^2^[2, N=108] 1.29, *p*=0.53).

 This was an almost identical response for the statement ‘research involves searching through sources such as libraries’ ( Figure 5) with no significant difference in response according to gender or KS (χ ^2^[2, N=2349] 8.23, *p*=0.02) and (χ ^2^[4, N=2538] 3.40, *p=*0.69) respectively ( Figure 4). The majority of respondents strongly agreed/agreed with this statement (81.5%). There was no statistical difference in how the pupils responded and how the teachers thought they would respond, (χ ^2^[2, N=108] 3.43, *p*=0.18).

**Figure 6.**
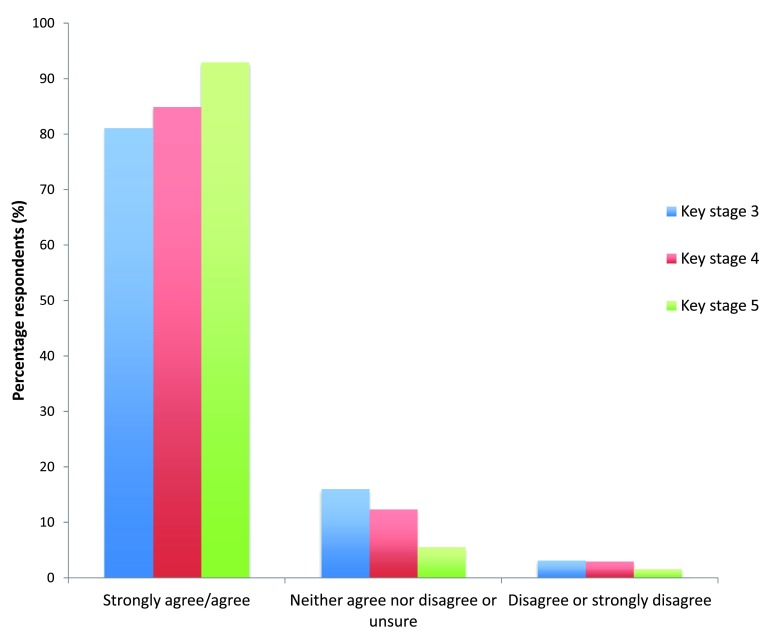
Percentage pupil responses across key stage to the statement ‘research can involve collecting data though interviews and questionnaires’. There was no significant difference in response with respect to gender (n=2368, *p*=0.42), but there was a significant difference across KS (n=2590, *p*<0.001).

Figure 6 shows that pupils clearly understand that research can involve collecting data through interviews and questionnaires. There was no significant difference in the way in which males and females responded to the statement ‘research can involve collecting data through interviews and questionnaires’ (χ ^2^[2, N=2368] 6.33, *p*=0.42). There was however a significant difference in the respondents across KS (χ ^2^[4, N=2590] 53.46, *p*<0.001). Pupils in KS5 (92.7%) are more likely to strongly agree/agree than KS4 (84.7%) and KS3 (80.9%) pupils. The majority of respondents strongly agreed/agreed with this statement (86.2%). This reflects the greater experience of research of KS5 pupils. There was no statistical difference in how the pupils responded and how the teachers thought they would respond, (χ ^2^[2, N=108] 2.61, *p*=0.27).

## The questionnaire data on pupil confidence in their research experience and ability

**Figure 7.**
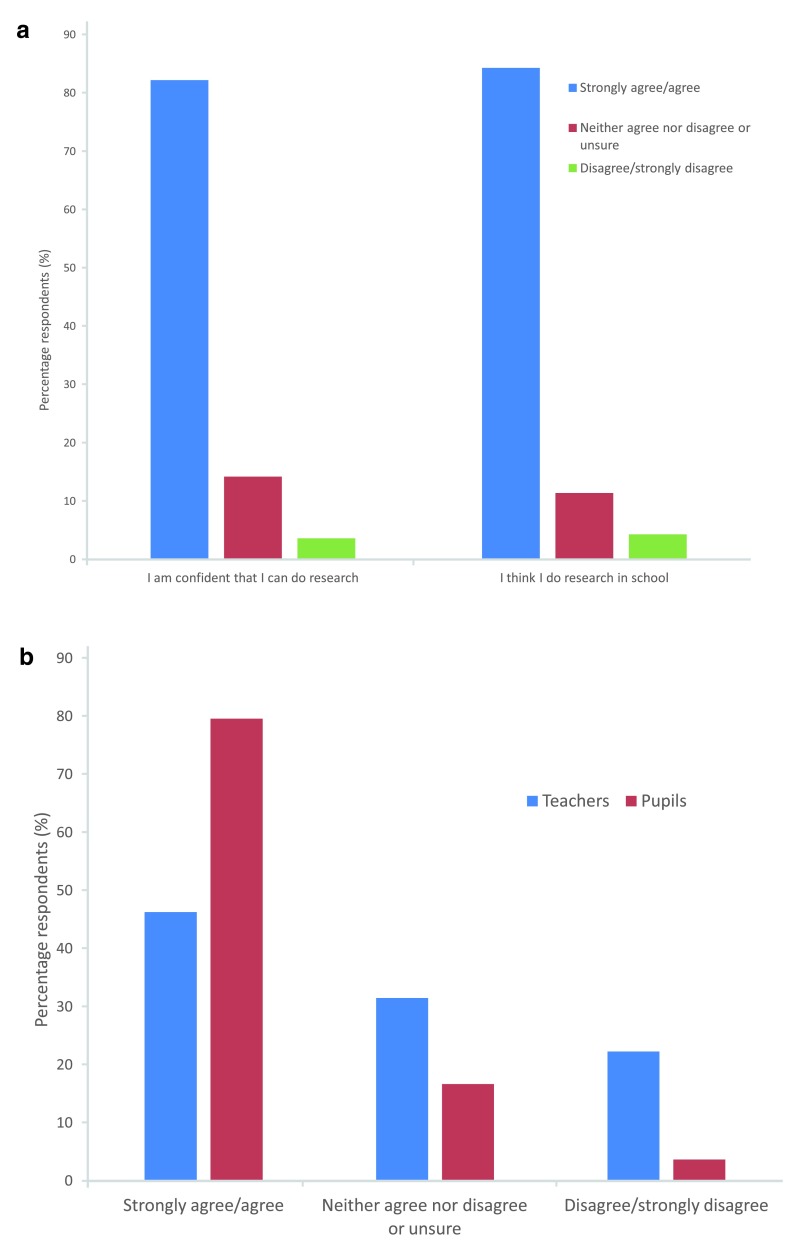
( **a**) Percentage of pupil responses to the ‘I am confident that I can do research’ and ‘I think I do research in school’. There was no significant difference with respect to gender (n=2373, *p*=0.09) or KS (n=2593, *p*=0.41) to the statement ‘I am confident that I can do research’. There was no significant difference with respect to gender (n=2349, *p*=0.007) or KS (n=2586, *p*=0.1) to the statement ‘I think I do research in school’. ( **b**) Comparison of the percentage distribution of responses from pupils and teachers to the statement ‘I am confident that I can do research’. There was a significant difference in how pupils and teachers responded to the statement (n=108, *p*=0.001).

Pupils are confident that they do research, and they think they do it in their school environment ( Figure 7a). For the statement ‘I am confident that I can do research’ there was no significant difference in response according to gender or across KS, (χ ^2^[2, N=2373] 4.89, *p*=0.09) and (χ ^2^[4, N=2593] 3.93, *p*=0.41) respectively. The majority of respondents (82.5%) strongly agreed/agreed with this statement. There was also no significant difference according to gender or across KS in responses to the statement ‘I think I do research in school’, (χ ^2^[2, N=2349] 9.88, *p*=0.007) and (χ ^2^[4, N=2586] 7.57, *p*=0.1) respectively ( Figure 7a). The majority of pupils (83.4%) strongly agreed/agreed with this statement.

There was however a significant difference in how the teachers thought pupils would answer this question (χ ^2^[2, N=108] 14.37, *p*=0.001). The teachers thought the pupils would be much less confident that they could do research ( Figure 7b). There was no significant difference between pupils and teachers on responses to the statement ‘I think I do research in school’ (χ ^2^[2, N=108] 1.40, *p*=0.49).

**Figure 8.**
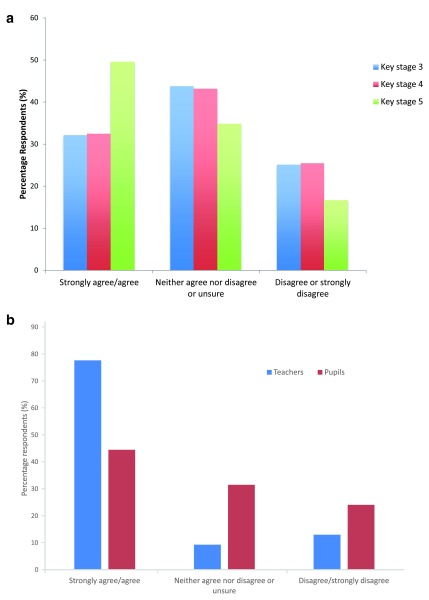
( **a**) Percentage pupil responses across key stage to the statement ‘doing research is challenging’. There was no significant difference in response with respect to gender (n=2367, *p*=0.38), but there was a significant difference across KS (n=2589, *p*<0.001). ( **b**) Comparison of the percentage distribution of responses from pupils and teachers to the statement ‘doing research is challenging’. There was no significant difference in response with respect to gender (n=2367, *p*=0.38), but there was a significant difference in how pupils and teachers responded to the statement (n=108, *p*=0.001).

As pupils progress through their educational experience, it is assumed that the work they are asked to do which involves research becomes more and more challenging. When asked to rate the statement ‘doing research is challenging’, there was a significant difference in the way in which pupils across KS responded ( Figure 8a). Pupils in KS5 were more likely to strongly agree/agree with this statement than those in either KS3 or 4. (χ ^2^[4, N=2589] 72.49, *p*<0.001). However, despite the assumed increase in challenging work, there was no significant difference between KS3 and 4 (χ ^2^[2, N=1748] 4.29, *p*=0.1). There was also no difference in how males and females responded to this statement (χ ^2^[2, N=2367] 1.94, *p*=0.38). There was however a significant difference in how teachers and pupils responded to this statement (χ ^2^[2, N=108] 13.25, *p*=0.001), with teachers thinking that pupils would find research challenging ( Figure 8b).

## The questionnaire data on pupil appreciation for research

**Figure 9.**
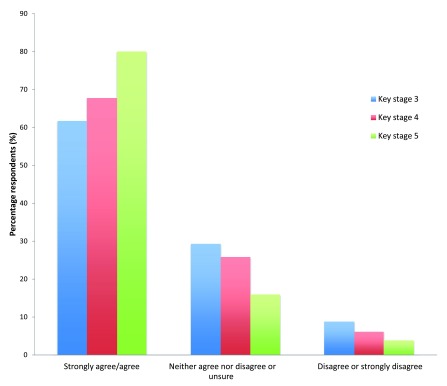
Percentage pupil responses across key stage to the statement ‘research is a worthwhile activity’. There was no significant difference in response with respect to gender (n=2370, *p*=0.006), but there was a significant difference across KS (n=2589, *p*<0.001).

In order for the UK to benefit in the future from a knowledge economy, pupils currently in school need to value research and think it of value to their careers. There was a significant difference in how pupils across KS responded to the statement ‘research is a worthwhile activity’ ( Figure 9) (χ ^2^[4, N=2589] 72.99, *p*<0.001). Pupils in KS5 were more likely to strongly agree/agree with this statement than KS3 (χ ^2^[2, N=1759] 72.92, *p*<0.001) or KS4 pupils (χ ^2^[2, N=1671] 48.70, *p*<0.001). There was also a significant difference in how KS3 versus KS4 pupils (χ ^2^[2, N=1748] 22.93, *p*<0.001) responded. This shows that as pupils progress through their education, they value research more. There was no significant difference in how males and females responded (χ ^2^[2, N=2370] 10.18, *p*=0.006).

There was no statistical difference in how the pupils responded and how the teachers thought they would respond, (χ ^2^[2, N=108] 0.43, *p*=0.81).

**Figure 10.**
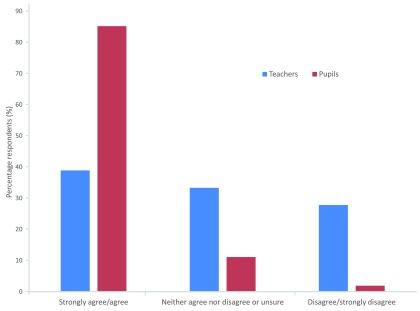
Comparison of the percentage distribution of responses from pupils and teachers to the statement ‘knowing how to do research will help my future career’. There was a significant difference in how pupils and teachers responded to the statement (n=108, *p*<0.001).

There was no significant difference in responses according to gender or across KS to the statement ‘knowing how to do research will help me in my future career (χ ^2^[2, N=2363] 6.59, *p*=0.04) and (χ ^2^[4, N=2584] 9.19, *p*=0.06) respectively ( Figure 10). The majority of respondents strongly agreed/agreed with this statement (76.9%). However, there was a significant difference in how teachers thought pupils would respond, with teachers thinking that pupils would not respond positively to this statement (χ ^2^[2, N=108] 27.57, *p*<0.001).

## Discussion

As stated in the introduction research can be defined in many different ways. Two of these three definitions include the word ‘new’ ( Redman & Mory, 1923; Research Excellence Framework http://www.ref.ac.uk/pubs/2011-02/), and this poses the following question: is research only about finding out new and original knowledge which is not known to anyone? Or can it also be applied to new knowledge not previously known to self, but known to others? The former is clearly the case for the REF where new, original research is judged. However, the latter scenario is often the case in schools, for example, where pupils are asked for homework to do ‘research’ in a particular area. This type of research is content driven ‘fact’ finding, the research question, or even just the topic often having been given as part of the homework task. Leedy & Ormrod (2010) describe this as ‘information discovery’ and do not consider it to be research. In terms of formal education, the NCSE only uses the term ‘research’ when linked to finding facts and using secondary sources. This is also clearly the case in exam board specifications, where pupils are required to conduct secondary research as part of controlled assessments. Thus the word ‘research’ can be applied to different scenarios of fact finding and data comparison. We feel that research as either new to ‘self’ or ‘new to all’ and thus original, is a crucial distinction in meaning, and colours how the term ‘research’ is both perceived and used by different groups of people, e.g. school pupils, teachers, government bodies, exam organisations, universities, as well as novice and experienced researchers. This is important because as pupils transition through their educational career the meaning and use of the word ‘research’ changes. One example of this is in higher education (HE) where leading universities are keen to promote their research-led teaching manifesto, where teaching is informed by research and research activity goes beyond fact finding and the investigation of secondary sources, and into novel enquiry and original investigation ( Yeoman & Zamorski, 2008).

Despite the specific linking of research in the NC to fact finding, and the lack of the use of the word ‘research’ in the NCSE at KS3 and 4, the learning outcomes of the NCSE do map onto the different research variations as outlined by Brew (2001). The mapping is dominated by domino variation (68%), that sees research categorised as task, activity, event, problems, technique and experiment. These scientific capabilities in pupils are important when demonstrating ‘scientific mastery’ as required by examination boards. Domino variation encompasses the concept of the scientific method, but while the steps of the ‘scientific method’ are referred to within the NC the actual term ‘scientific method’ is not present. Instead the phrase ‘working scientifically’ is used to describe “ *the key features of science enquiry, so that pupils learn to answer relevant scientific questions*” (p.169) ( https://www.gov.uk/government/uploads/system/uploads/attachment_data/file/335116/Master_final_national_curriculum_220714.pdf.). Whilst many scientists struggle to recognise the step-by-step scientific method as portrayed by Keeslar (1945) in how they conduct their research, what is not in contention is that research must begin with a sensible question. This question can arise from ongoing observation and experimentation, or it might come from a systematic review of existing research. Despite the fact that “ *asking questions*” appears in the NCSE, Reiss (2015) states that “ *we don’t do a very good job of getting pupils in school science lessons to ask the sorts of questions that scientists actually ask*”. This study provides evidence that less than 40% of secondary school pupils thought that it was necessary to start research with a question. In order to ascertain if a research question is worth pursuing, then background information must be gathered to see if answers to the question already exist, or if the question needs to be refined in the context of what is already known. However, in the school environment, the search for background information is often divorced from the actual question setting thus the whole picture of scientific enquiry cannot emerge. Initiatives such as the Extended Project Qualification (EPQ) AS- level and the new GCSE equivalent, will help with this issue, and allow pupils to experience full scientific enquiry. The EPQ is a dissertation or investigation/field study which involves establishing and then addressing a research question through either a literature review and argumentative discussion or data collection and analysis. In the 2014–15 academic year 33,564 pupils completed the EPQ ( http://www.jcq.org.uk/examination-results/a-levels/2015/a-as-and-aea-results-summer-2015). As Malcolm Trobe (Deputy General Secretary of the Association of School and College Leaders) stated in a recent BBC article ( http://www.bbc.co.uk/news/education-33819871).

" *(EPQs) are phenomenally valuable in giving young people the opportunity to prepare themselves for university where they will spend much of their time studying and learning through their own research and reading*."

One of the other issues we raised in this paper is that of being unbiased during systematic investigation. Only 16.8% of pupils disagreed/strongly disagreed, with the statement ‘you do research to confirm your own opinion’. One of the core premises of the scientific method is that researchers remain unbiased and the NCSE at all key stages requires pupils to “ *pay attention to objectivity*” (p.4) ( https://www.gov.uk/government/uploads/system/uploads/attachment_data/file/335174/SECONDARY_national_curriculum_-_Science_220714.pdf). Confirmation bias is a well-known phenomenon and it is where researchers (including scientists) tend to look for and only see evidence that confirms what they already believe ( Nickerson, 1998).

Only 32% of pupils found research challenging at KS3 and 4, but this increased to 49% at KS5. This reflects the increased complexity of the material taught at A level and the requirement for more critical analysis of sources. This can be mapped to the ‘layer variation’ of Brew’s framework. Learning outcomes linked to layer variation are poorly represented at KS3, but increase at KS4 and 5. KS5 provides the chance to do more sophisticated practicals and fieldwork, as well as the opportunity to do qualifications such as the EPQ (Level 3). It is perhaps surprising not to see an increase in percentage between KS3 and 4, suggesting that teachers could challenge pupils more at KS4 in terms of scientific enquiry. This could be resolved by the introduction of Level 2 project qualification such as that offered by the AQA exam board ( http://www.aqa.org.uk/subjects/projects/aqa-certificate/PQ2-7992/spec-at-a-glance). These initiatives could also help with increasing the ‘trading variation’ linked learning outcomes, which are the least represented within the NCSE with only 11%. Trading variation is where research is categorised as ‘product and social phenomenon’, and would include publication and the presentation of results. Project qualifications include an assessment of an oral presentation, and the dissertations could be prepared for publication in school journals and magazines. Another interesting finding linked to this is that teachers think that pupils will find research more challenging than perhaps they do. Again this may come down to the perception of the term research. The majority of teachers are graduates, with HE research experience; when teachers set pupils homework tasks to ‘research’ a topic, they may be using the word ‘research’ in a different way to how they would actually define it, thus leading to the disparity seen in this study.

The UK has a knowledge economy dependent upon science and research. Thus we have a need for STEM subjects to be taught in schools and to encourage the new workforce to take STEM subjects to match STEM need in the future. This pipeline requires pupils to understand the range of careers which require STEM subjects. However the analysis of the learning outcomes of the NCSE show that only 19% of them map to ‘journey variation’ which is linked to growth and transformation. As part of the Education Act of 2011, the government placed responsibility for career guidance into individual schools, rather than it being provided by local authorities or central government. However, there was no funding and no guidance on how this should be achieved ( Hooley *et al.*, 2012). In a review, ‘Career 2020’, jointly written by the Pearson Think Tank and International Centre for Guidance Studies at the University of Derby, Hooley *et al*. recommend that we encourage schools to think of careers as being “ *a key component of their mission and to actively link this to the curriculum*” (p.4) ( http://derby.openrepository.com/derby/bitstream/10545/251032/1/CAREERS+2020.pdf). There is evidence which suggests that this approach of linking careers to the curriculum is the most effective, but requires considerable buy-in from school senior leadership teams. It is stated in the KS5 NCSE that pupils should “ *develop an interest in further study and careers associated with the subject*” (p.3) ( https://www.gov.uk/government/uploads/system/uploads/attachment_data/file/446829/A_level_science_subject_content.pdf), this outcome however is missing in the NCSE for KS3, and only briefly mentioned at KS4 where is states that teaching should “ *establish the basis for a wide range of careers*” (p.3) ( https://www.gov.uk/government/uploads/system/uploads/attachment_data/file/381380/Science_KS4_PoS_7_November_2014.pdf).

This research presented here suggests that pupils think that research will be valuable to them in their future career, although it was also clear that teachers did not think that pupils would value this as much as they did. As discussed earlier this may be due to the perception of the term ‘research’. Pupils also think research is a worthwhile activity, and this positive feeling increases during their educational career, possibly as they are exposed to more opportunity, e.g. through the EPQ. These positive views are examples of how research is seen as ‘journey’ where activity enables growth and transformation within the Brew (2001) framework. We are now seeking more nuanced and elaborate pupil perceptions through the analysis of focus group interviews that we conducted after the questionnaire.

Finally, Brew (2001) suggests that the framework would be a useful tool to evaluate research performance by individuals, but we have also found that it provides a framework to map curricula.

## Data availability

The data referenced by this article are under copyright with the following copyright statement: Copyright: © 2016 Yeoman K et al.

Data associated with the article are available under the terms of the Creative Commons Zero "No rights reserved" data waiver (CC0 1.0 Public domain dedication). 

F1000Research: Dataset 1. Complete pupil data set, 10.5256/f1000research.7449.d108247 ( Yeoman *et al*., 2015a).

F1000Research: Dataset 2. Compiled teacher: pupil data set, 10.5256/f1000research.7449.d108248 ( Yeoman *et al*., 2015b).

## References

[ref-1] Brew A: Conceptions of Research: a phenomenographic study. *Stud High Educ.* 2001;26(3):271–285. 10.1080/03075070120076255

[ref-2] Brookdale Consulting:. Impact of the Institute of Food Research.2013 Reference Source

[ref-3] Donghong C Shunke S: The more, the earlier, the better: science communication supports science education.editors. Cheng D, Claessens M, Gascoigne T. Springer;2010.

[ref-4] Fennema E Sherman JA: Fennema-Sherman Mathematics Attitudes Scales: Instruments designed to measure attitudes toward the learning of mathematics by females and males. *J Res Math Educ.* 1976;7(5):324–326. 10.2307/748467

[ref-5] Hooley T Marriot J Watts AG: Careers 2020: Options for future careers work in English schools.Pearson.2012 Reference Source

[ref-6] International Comparative Performance of the UK Research Base. In: Department of Business IaS. editor. Elsevier;2013 Reference Source

[ref-7] Keeslar O: The elements of scientific method. *Sci Educ.* 1945;29(5):273–8. 10.1002/sce.3730290512

[ref-8] Leedy PD Ormrod JE: Practical Research: Planning and Design.Ninth Edition, published by Merrill. Pearson Education, Inc.2010 Reference Source

[ref-9] McComas WF: The Nature of Science in Science Education: Rationales and Strategies. Kluwer Academic Publishers;1998;53–70.

[ref-10] Meyer JH Shanahan MP Laugksch RC: Students’ Conceptions of Research. I: A qualitative and quantitative analysis. *Scand J Educ Res.* 2005;49(3):225–244. 10.1080/00313830500109535

[ref-11] Nickerson RS: Confirmation Bias: A Ubiquitous Phenomenon in many guises. *Rev Gen Psychol.* 1998;2(2):175–220. Reference Source

[ref-12] Purver M: The Royal Society: Concept and Creation, Routledge Library Editions: History and Philosophy of Science.Routledge,2013;25.

[ref-13] Redman LV Mory AVH: The Romance of Research. The Williams and Wilkins Company;1923.

[ref-14] Reiss MJ: The Nature of Science. In: *Learning to Teach Science in the Secondary School: A Companion to school experience.*editor, Toplis R,2015;64–73. Reference Source

[ref-15] Sarma GP: The Art of Memory and the Growth of the Scientific Method. Cornell University Library, arXiv: 1307.0254 (physics.hist-ph).2014 Reference Source

[ref-16] The Oxford English Dictionary. "research, *n.*"OED online, Oxford University Press. Reference Source

[ref-17] Wikoff RL Buchalter BD: Factor analysis of four Fennema-Sherman mathematics attitude scales. *Int J Math Educ Sci Technol.* 1986;17(6):703–706. 10.1080/0020739860170605

[ref-18] Yeoman KH Zamorski B: Investigating the impact on skill development of an undergraduate scientific research skills course. *beej.* 2008;11:5 Reference Source

[ref-19] Yeoman K Bowater L Nardi E: Dataset 1 in: The representation of research in the National Curriculum and secondary school pupils’ perceptions of research, its function, usefulness and value to their lives. *F1000Research.* 2015a Data Source 10.12688/f1000research.7449.1 PMC4722701 26835002

[ref-20] Yeoman K Bowater L Nardi E: Dataset 2 in: The representation of research in the National Curriculum and secondary school pupils’ perceptions of research, its function, usefulness and value to their lives. *F1000Research.* 2015b Data Source 10.12688/f1000research.7449.1 PMC4722701 26835002

