# Supplementary material for: The representation of scientific research in the national curriculum and secondary school pupils’ perceptions of research, its function, usefulness and value to their lives
Source: F1000Res. 2016 Feb 12;4:1442. Originally published 2015 Dec 14. [Version 2] doi: 10.12688/f1000research.7449.2 (PMC4722701; doi:10.12688/f1000research.7449.2)
Supplement: Supplementary file 3 [file f1000research-4-8589-s0002.tgz › 64924f73-e7e8-4223-930b-250e28a5e71c.docx]

[
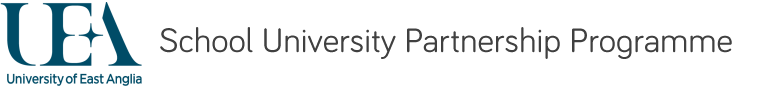
](http://ueasupp.org/)

| Male 🞎 | Year 7 🞎 | Year 10 🞎 | Year 12 🞎 | ***We thank you very much for taking the time to help us with our research!*** *Kay Yeoman, Project Director* |
| --- | --- | --- | --- | --- |
| Female 🞎 | Year 8 🞎 | Year 11 🞎 | Year 13 🞎 |  |
|  | Year 9 🞎 | State School 🞎 | Independent School 🞎 |  |
|  | | | |  |
| This short questionnaire aims to explore your views on what is research, who uses it, how it is conducted, whether you see it as something useful and enjoyable, and as something that you are good at and interested in. We expect this to take no longer than 15 minutes to complete. | | | | |

Please shade the box 1, 2, 3, 4 or 5, with **1** standing for **Strongly Agree** and 5 for **Strongly Disagree**.

Shade 3 if you neither agree nor disagree, or if you are unsure.

|  | **Statement** | **1** | **2** | **3** | **4** | **5** |
| --- | --- | --- | --- | --- | --- | --- |
| 1. | Scientists do a lot of research. | 🞎 | 🞎 | 🞎 | 🞎 | 🞎 |
| 2. | Research is a worthwhile activity. | 🞎 | 🞎 | 🞎 | 🞎 | 🞎 |
| 3. | Knowing how to do research will help me in my future career. | 🞎 | 🞎 | 🞎 | 🞎 | 🞎 |
| 4. | People around me would not take me seriously if I said I was interested in a career in research. | 🞎 | 🞎 | 🞎 | 🞎 | 🞎 |
| 5. | Research will not be important in my life's work. | 🞎 | 🞎 | 🞎 | 🞎 | 🞎 |
| 6. | I am confident that I can do research. | 🞎 | 🞎 | 🞎 | 🞎 | 🞎 |
| 7. | Historians do a lot of research. | 🞎 | 🞎 | 🞎 | 🞎 | 🞎 |
| 8. | Doing research is challenging. | 🞎 | 🞎 | 🞎 | 🞎 | 🞎 |
| 9. | Research can be carried out through collecting data during a fieldtrip. | 🞎 | 🞎 | 🞎 | 🞎 | 🞎 |
| 10. | Artists do a lot of research. | 🞎 | 🞎 | 🞎 | 🞎 | 🞎 |
| 11. | You have to be a genius to do research. | 🞎 | 🞎 | 🞎 | 🞎 | 🞎 |
| 12. | Research involves coming up with new theories. | 🞎 | 🞎 | 🞎 | 🞎 | 🞎 |
| 13. | The main purpose of research is to generate new knowledge. | 🞎 | 🞎 | 🞎 | 🞎 | 🞎 |
| 14. | Research involves collecting new data. | 🞎 | 🞎 | 🞎 | 🞎 | 🞎 |
| 15. | Research always involves investigating a question. | 🞎 | 🞎 | 🞎 | 🞎 | 🞎 |
| 16. | Research involves searching through sources, such as libraries. | 🞎 | 🞎 | 🞎 | 🞎 | 🞎 |
| 17. | Philosophers do a lot of research. | 🞎 | 🞎 | 🞎 | 🞎 | 🞎 |
| 18. | Doing research is not useful. | 🞎 | 🞎 | 🞎 | 🞎 | 🞎 |
| 19. | Research can involve collecting data through interviews and questionnaires. | 🞎 | 🞎 | 🞎 | 🞎 | 🞎 |
| 20. | You do research to confirm your own opinion. | 🞎 | 🞎 | 🞎 | 🞎 | 🞎 |
| 21. | Lawyers do a lot of research. | 🞎 | 🞎 | 🞎 | 🞎 | 🞎 |
| 22. | Research is carried out solely through experiments in a laboratory. | 🞎 | 🞎 | 🞎 | 🞎 | 🞎 |
| 23. | Anybody can do research. | 🞎 | 🞎 | 🞎 | 🞎 | 🞎 |
| 24. | Mathematicians do a lot of research. | 🞎 | 🞎 | 🞎 | 🞎 | 🞎 |
| 25. | I think I do research in school. | 🞎 | 🞎 | 🞎 | 🞎 | 🞎 |
