# Supplementary material for: The representation of scientific research in the national curriculum and secondary school pupils’ perceptions of research, its function, usefulness and value to their lives
Source: F1000Res. 2016 Feb 12;4:1442. Originally published 2015 Dec 14. [Version 2] doi: 10.12688/f1000research.7449.2 (PMC4722701; doi:10.12688/f1000research.7449.2)
Supplement: Peer review history

# Peer review: Referee response for version 2

**DOI:** 10.5256/f1000research.8589.r12429

**Published:** 2016-02-15

**Authors:** Beatrix Fahnert

**Affiliations:** 1 Cardiff School of Biosciences, Cardiff University, Cardiff, UK

The revised version evidences that the authors thoroughly considered the reviewer comments.

I have read this submission. I believe that I have an appropriate level of expertise to confirm that it is of an acceptable scientific standard.

# Peer review: Referee response for version 1

**DOI:** 10.5256/f1000research.8026.r11953

**Published:** 2016-01-18

**Authors:** Joanna Verran, James Redfern

**Affiliations:** 1 School of Research, Enterprise and Innovation, Manchester Metropolitan University, Manchester, UK

In our opinion, this is a very useful output. The mapping of student and teacher responses against the representation of research within the English national curriculum provides a robust comparison, and the description of the perceptions of students as to the meaning of research is really interesting.

The title might better describe the study if it specifically says ‘scientific’ research instead of research. Whilst the authors discuss the other uses of the word research, the questions and answers are specifically in relation to scientific research.

We do not particularly like ‘it’s’ in the first line of the abstract. We would also like to see a more clear concluding sentence in the abstract.

The study is thorough and the number of participants impressive. It would be interesting to note if all students were taking science, particularly at KS5. We assume they were. Were all the teachers Science teachers?

Perhaps a little information within the introduction, as to what other sections of society think of research, might be useful. What do non-scientist adults think for example? There are some interesting reports on this that may help the reader understand the results within the context of society.

The mapped curriculum using the Brew descriptors is very interesting. However, the results need more discussion: currently there is very little to describe what they mean, their context, and what, if any, conclusions can be drawn from that analysis. 

Can the authors evidence their assumption that students have a greater understanding of meta-analysis of data by KS5 than earlier on in their school journey? Or is it a speculative comment. This could be made clearer.

How do the findings about the practice of ‘research’ in schools relate to the ‘nature of science’ debate? We also wonder how findings from this study would compare to findings using undergraduates at different stages in their degrees?

We have read this submission. We believe that we have an appropriate level of expertise to confirm that it is of an acceptable scientific standard.

# Response

**Published:** 2016-01-19

**Authors:** Kay Yeoman

**Affiliations:** University of East Anglia, UK

Thank you for the positive comments and suggestions for improvement. We will be revising and then submitting a new version in the light of your review.

# Peer review: Referee response for version 1

**DOI:** 10.5256/f1000research.8026.r11568

**Published:** 2015-12-21

**Authors:** Beatrix Fahnert

**Affiliations:** 1 Cardiff School of Biosciences, Cardiff University, Cardiff, UK

Supporting the development of a realistic perception of research by pupils is crucial in our aims to inspire the next generations of scientists, and to scientifically engage well-informed next generations of the public at a level necessary for the benefit of everyone (e.g. in context of economy, healthy lifestyle, openness towards technology, sustainability) and having been enabled to evaluate relevant pros and cons when making decisions. Therefore, it is necessary to understand current perceptions, how they arise, how they may change and how they relate to the pupils' engagement with research. An analysis of how the concept of research is embedded in the curriculum, whether research is portrayed as valuable and accessible, and how the educational environment affects pupils' perceptions of research allows to constructively suggest ways forward in the quest.

The authors shall be thanked for this important contribution to the field.

The aims of the research are clear and well supported by methodology (fully ethically cleared). The questionnaires were piloted and optimised before the large survey.

The Abstract summarises the article well.

The Introduction sets the scene very well, and the Materials and Methods section was mostly clear including the parameters of the institutions.

The very interesting findings are well presented in the Results section. For instance, Table 4 provides a clear mapping of learning outcomes at key stages against the used framework. It visualises how perception and understanding of research should progress through key stages. This then is found in the questionnaire data (e.g. pupils value research more as they progress through education).

How research is referred to/not referred to in the curriculum and by exam boards is an interesting finding. The authors also emphasise issues around the use of the term ‘research’ (fact finding vs. novel enquiry) and related consequences in context of e.g. preparing pupils for transition into HE, pupils' understanding of the notion of research-led teaching when selecting their future HEI.

A further issue highlighted by the authors is the need for pupils to better understand that research should be unbiased, that a research question is the starting point and how a sound research question is arrived at (and how e.g. the new Extended Project Qualification may support an improved understanding).

Teachers were found to mostly correctly gauge their pupils' perception except for whether pupils feel they do research in school, whether pupils find research challenging and whether research is seen as useful by pupils for their future career (importantly here: pupils indeed see the benefit).

An encouraging (women in STEM context) finding is that there is no difference in perception between genders.

I would like to approve the article and to suggest some minor changes to be made in a revision of the current manuscript.

- The authors have already provided a contextual discussion of their findings, but have not yet discussed the potential impact of the responders on the data or explained how such impact may have been limited. This needs to be added, because responses are likely provided by a self-selecting group (e.g. 2634 from 6000 pupils; teachers [does the subject background matter?]; institutions already being part of the project). There is also a statement that the study is part of the students’ learning experience about research. How might this have affected responses?
- I think it would add further value to the figures and make them more self-explanatory if n-values were added as well as a visual indication if there are significant differences in responses; and adding figure legends that state related findings such as response trends (without the actual data, which remain reported in the text as they already are) regarding gender, KS, teacher responses.
- Some general indication of how 'agree' and 'disagree' responses compare with the reported 'strongly agree/ disagree'/'unsure' would be useful.

 

There are two further points regarding the Materials and Methods section, which the authors may also wish to address when making changes to the current version of the manuscript.

- The words "and the interview audio recordings and transcripts" could be removed to avoid confusion, because these are unrelated to the reported analysis and only relate to subsequent work.
- The section "The representation of research in the national curriculum for science in England and the examination board specifications covering the mapping of the national curriculum" could be part of Materials and Methods rather than Results.

I have read this submission. I believe that I have an appropriate level of expertise to confirm that it is of an acceptable scientific standard.

# Response

**Published:** 2016-01-30

**Authors:** Kay Yeoman

**Affiliations:** University of East Anglia, UK

Thank you for your useful comments on our paper. We have taken your points into consideration in the revised manuscript and it has improved as a result. 

**The authors have already provided a contextual discussion of their findings, but have not yet discussed the potential impact of the responders on the data or explained how such impact may have been limited. This needs to be added, because responses are likely provided by a self-selecting group (e.g. 2634 from 6000 pupils; teachers [does the subject background matter?]; institutions already being part of the project). There is also a statement that the study is part of the students’ learning experience about research. How might this have affected responses?**

Response: We have clarified some of these points in responses to the other referee’s comments and including some more clarification in the text. The schools taking part in the project were our partner schools from the RCUK School:University Partnership Initiative (SUPI). A range of schools were chosen for the SUPI on their existing contact with the University and being the type of school which reflected those in Norfolk (e.g. city, rural, coastal). Pupils were not self-selecting on answering the questionnaire, we were dependent on the form teachers conducting the questionnaire during form time and then handing the completed questionnaires back to the link teacher. The teachers responding to the questionnaire would have been from a variety of subject disciplines across the science and the humanities. Yes, this study and the SUPI project is about embedding research activity into the school environment. The questionnaire was conducted as a baseline before the SUPI activities had really been established within the schools.

**I think it would add further value to the figures and make them more self-explanatory if n-values were added as well as a visual indication if there are significant differences in responses; and adding figure legends that state related findings such as response trends (without the actual data, which remain reported in the text as they already are) regarding gender, KS, teacher responses.**

****

Response: We totally agree, and this has been added to the Figure legends. We have included more information in the figure legends. As well as the n= we also included the *p *value, but the detailed statistic reporting is still in the text.

**Some general indication of how 'agree' and 'disagree' responses compare with the reported 'strongly agree/ disagree'/'unsure' would be useful.**

Response: Likert scales are subject to distortion; respondents may avoid extreme responses. The data set presented here is large, and yes there were some differences in the responses to statements between strongly agree/agree as well as strongly disagree/disagree. For example more pupils at KS5 (28.4%) strongly agreed that research is worthwhile activity than either KS3 (19.2%) or 4 (19.1%), but this outcome of a higher percentage of KS5 pupils agreeing with this statement did not change post consolidation of the strongly/agree and agree responses. After consolidation there was a significant difference between KS3 and 4, not apparent when just looking at the strongly agree data alone.  This was because far more KS4 pupils (52%) agreed with this statement that KS3 pupils (42%). 

In our view the consolidation of strongly agree/agree as well as strongly disagree/disagree allowed for a more straightforward statistical analysis using the Chi Squared test and a clearer reporting of the findings. However we do take the point that we may be missing more nuanced findings within the data set by taking this strategy.

**There are two further points regarding the Materials and Methods section, which the authors may also wish to address when making changes to the current version of the manuscript.**

**The words "and the interview audio recordings and transcripts" could be removed to avoid confusion, because these are unrelated to the reported analysis and only relate to subsequent work.**

Response: We agree and this has been deleted

**The section "The representation of research in the national curriculum for science in England and the examination board specifications covering the mapping of the national curriculum" could be part of Materials and Methods rather than Results.**

Response: It is interesting that you mentioned this, as we considered having it there, but decided against as we felt that this was original analysis and a ‘result’ rather than a method.
